# Revolutionizing ESCC prognosis: the efficiency of tumor-infiltrating immune cells (TIIC) signature score

**DOI:** 10.1007/s12672-024-01709-3

**Published:** 2025-01-20

**Authors:** Haixia Wang, Shaowei Ma, Zixin Yang, Ren Niu, Haiyong Zhu, Shujun Li, Shaolin Gao, Zhirong Li, Yanhua Tian

**Affiliations:** 1 Department of Radiation Oncology, The Fifth Clinical Medical College of Henan University of Chinese Medicine, Zhengzhou People’s Hospital, Zhengzhou, 450003 China; 2 Department of Gastrointestinal Surgery, The Second Hospital of Hebei Medical University, Shijiazhuang, 050000 China; 3 Second Department of Oncology, The Second Hospital of Hebei Medical University, Shijiazhuang, 050000 China; 4 Department of Thoracic Surgery, The Second Hospital of Hebei Medical University, Shijiazhuang, 050000 China; 5 Clinical Laboratory Center, The Second Hospital of Hebei Medical University, Shijiazhuang, 050000 China

**Keywords:** Tumor-infiltrating immune cells (TIIC), Prognosis, Tumor microenvironment, Esophageal squamous cell carcinoma (ESCC)

## Abstract

**Background:**

Patients suffer from esophageal squamous cell carcinoma (ESCC), which is the ninth highly aggressive malignancy. Tumor-infiltrating immune cells (TIIC) exert as major component of the tumor microenvironment (TME), showing possible prognostic value in ESCC.

**Methods:**

Transcriptome data and scRNA-seq data of ESCC samples were extracted from the GEO and TCGA databases. Tissue Specific Index (TSI) was defined to identify potential TIIC-RNAs from the TME. Twenty machine learning algorithms were further applied to evaluate the prognostic efficacy of TIIC signature score. Gene colocalization analysis was performed. Differences in CNV on chromosomes and SNP sites of prognostic model genes were calculated.

**Results:**

The most reliable model of TIIC signature score was developed based on three prognostic TIIC-RNAs. It showed a higher C-index than any other reported prognostic models. ESCC patients with high TIIC signature score showed poorer survival outcomes than low TIIC signature score. The activity of most immune cells decreased with the increase of TIIC score. TIIC signature score showed difference in the expression levels and methylation levels of DEGs. There was also significant different correlation with the degree of CNV amplification and CNV deletion of the immune checkpoint genes. Gene colocalization analysis showed two prognostic model genes (ATP6V0E1 and BIRC2). MR analysis found that rs148710154 and rs75146099 SNP sites of TIIC-RNA gene had a significant correlation between them gastro-oesophageal reflux and ESCC.

**Conclusion:**

TIIC signature score was the first time developed which provided a novel strategy and guidance for the prognosis and immunotherapy of ESCC. It also gave the evidence in the important role of immune cells from the TME in the treatment of cancers.

## Introduction

Esophageal squamous cell carcinoma (ESCC) is a prevalent malignant tumor within the digestive system [1, 2]. Despite the potential treatment options of esophagectomy combined with chemotherapy and radiation therapy, the prognosis for ESCC remains generally unfavorable, with a relatively low 5-year relative survival rate [3]. The identification of genomic variations in cancer cells is widely recognized as a significant factor contributing to the advancement of ESCC. The detection of efficacious genomic signatures holds paramount importance to promote the therapeutic outcomes of ESCC. It is acknowledged that the TNM staging system has been widely utilized in clinical decision-making, but the inherent molecular heterogeneity of the disease affects the accuracy of TNM [4]. Consequently, there is an urgent need for genomic signatures with robust clinical significance for ESCC to supplement the TNM staging system and offer more accurate assessment and efficacy of prognosis and therapy.

There is a growing number of evidence highlighting the significance of the tumor microenvironment (TME) within ESCC tissues [5, 6]. The TME encompasses various cellular components, including vascular endothelial cells, immune-infiltrating cells, and stromal cells, which exert both bifacial effects [6]. The presence of multiple immune cells is often associated with cancer metastasis and unfavorable disease prognosis [7]. Notably, diminished T cells and NK cells have been identified as the primary cellular constituents in ESCC [8]. Tumor-associated macrophages may contribute to a stimulative role by the immune evasion mechanisms [9]. A recent study has examined the relationship between prognostic markers for ESCC and immune cells [10]. In ESCC patients, the number of NK cells and macrophages has been found to have a significant impact on postoperative prognosis. Macrophages located in the TME can undergo polarization into either tumor-suppressive M1 or pro-inflammatory M2 phenotypes [11]. Furthermore, the upregulation of marker gene in tumor tissues has been associated with the polarization of M0 macrophages towards the M2 phenotype and subsequent infiltration into the TME, leading to a poor prognosis for patients with ESCC [12]. Therefore, gaining a comprehensive understanding of the TME status in ESCC patients could provide valuable insights into their genomic profiles and potentially enhance their prognosis.

Single-cell RNA sequencing (scRNA-seq) represents a remarkable opportunity to investigate the intra-tumor heterogeneity of ESCC and its TME, thereby addressing these research questions. In recent years, multiple studies have successfully gained insights into the collective behavior of tumor cells in ESCC by employing this technique on human ESCC primary tumor tissues and their adjacent normal tissues [13]. The advent of machine learning has also emerged as a prospective remedy for the problem in the creation of prognostic models based on intricate clinical data [14]. Machine learning is an interdisciplinary field that combines computer science and computational statistics to improve the effectiveness of disease prognosis and therapeutic decision-making procedures [15]. In this study, we analyze the transcriptome of individual cells derived from ESCC patients in order to develop a novel tumor-infiltrating immune cell (TIIC)-related risk model for the prognosis of ESCC based on multiple machine learning algorithms.

## Materials and methods

### Data source

scRNA-seq dataset was derived from GSE160269 in the GEO database, containing 60 ESCC samples. Bulk RNA-seq profiles and corresponding clinical data of ESCC patients (n = 81) were obtained from TCGA database. The ESCC chip data of GEO database included GSE53622 (n = 60), GSE53624 (n = 119) and GSE53625 (n = 179) used. The normalizeBetweenArrays function in the “limma” R package was conducted for data correction of the chip data. The TCGA cohort was utilized for analyzing the proportions of different cell types and establishing the prognostic model, while the GEO cohort served as the validation set for the prognostic model.

### Processing scRNA-seq data

We performed the “Seurat” R package (v4.1.3) for analysis. Cells that did not meet the specified quality control criteria were eliminated from the integrated dataset. The mitochondrial content < 10%, and the limit range of UMI count: 200–50000 and gene count: 200–6000. We used the FindAllMarkers function to calculate differentially expressed genes (DEGs) between clusters or cell types with *p* < 0.05, log_2_FC > 0.25, and a ratio of expression > 0.1. Batch effects were processed using harmony. The “survminer” R package was conducted to determine the best cutoff value.

### Identification of TIIC-related genes

The novel computational framework of TIIC signature score was developed based on immune cells and tumor cells at single-cell level and bulk RNA level of ESCC tissues.

First, the top 15% RNAs with high expression levels were considered as potentially immune-related RNAs.

Second, tissue specific index (TSI) was defined to identify potential immune-related RNAs by.

                                              TSI_RNA_ =  $$\frac{{\sum }_{i=1}^{N}(1-{x}_{RNA,i})}{N-1}$$

N refers to the immune cell type and the amount of xRNA, and i refers to the RNA expression intensity in each immune cell based on the normalized maximum value. 0 < TSI < 1. Immune cell universal RNA is defined when TSI equals 0 and immune cell specific RNA is defined when TSI equals 1. RNAs with high expression are classified as immune-related universal RNAs (iuRNAs). Significantly up-regulated in immune cell types and down-regulated in tumor cells iuRNAs are defined as TIIC-RNAs.

Third, we screened most valuable TIIC-RNA by applying Boruta, limit gradient lift (Xgboost), minimum absolute contraction and selection operator regularized logistic regression (LassoLR), support vector machines (SVM), random forest (RF) and microarray predictive analysis (Pamr) algorithms.

### Cell annotation analysis

Cell annotation analysis identified epithelial cell marker, fibroblast marker, endothelial cell marker, T cell marker, NK cell marker, B cell marker, myeloid cell marker, and mast cell marker. In addition, we separated immune cell clusters and annotated each cell types by Sc-Type software.

### Functional analysis of TIIC signature score

We quantified 6 immune infiltrating cells by tumor immune estimation resource (TIMER) algorithm, 28 immune cells by the single cell gene set enrichment analysis (ssGSEA) algorithm, and 10 immune cells by the microenvironment cell populations-counter (MCPcounter) algorithms, as well as ESTIMATE algorithms. The gene ontology (GO) and kyoto encyclopedia of genes and genomes (KEGG) analyses were conducted. Metascape was used for enrichment analysis. Metabolic pathways in the KEGG database were also quantified using gene set variation analysis (GSVA).

### Prediction of immunotherapy response

Immunotherapy response was predicted with Nathanson (melanoma), GSE35640 (melanoma), GSE91061 (melanoma), GSE78220 (melanoma), IMvigor210 (uroepithelial carcinoma, UC), Braun (renal cell carcinoma, renal cell carcinoma). RCC), GSE179351 (colorectal adenocarcinoma and pancreatic adenocarcinoma, COAD and PAAD), GSE165252 (esophageal adenocarcinoma, ESCA), GSE103668 (triple-negative breast cancer, TNBC) and GSE126044 (non-small cell lung cancer, NSCLC) datasets. TIIC signature scores were calculated for each dataset to predict immunotherapy response. At the same time, we also took TIDE online tools (http://tide.dfci.harvard.edu/) to predict the immune response score in the TCGA dataset.

### Gene set enrichment analysis (GSEA)

In order to estimate the functional characteristics of TIIC groups, we performed “limma” R package to analyze the expression level between two groups at the threshold of *p* < 0.05 and abs (log_2_FC) > 0.5. We utilized the “clusterProfiler” R package to conduct GSEA analysis in up-regulated genes. The related gene sets of KEGG and GO-BP were from the MSigDB database. When the *p* value was less than 0.05 after BH correction, the enrichment function was obtained based on “enrichplot” R package.

### Genomic variation landscape

The “maftools” R package was manipulated to identify the top 30 mutated genes between two score groups. Chi-square tests were performed to analyze mutation frequencies of mutated genes between high-risk and low-risk groups. CNV data was processed using Gistic 2.0 software. Subsequently, we assessed significantly amplified and deleted chromosome segments and differences in CNV on chromosomes. The fraction of genome alteration (FGA), the fraction of genome gained (FGG), and the fraction of genome lost (FGL) were calculated. Finally, the “ggplot2” R package was utilized to visualize these CNV results.

### Construction of prognostic TIIC-related risk model generated by machine learning methods

Univariate Cox regression analysis was performed to screen candidate prognostic TIIC-RNA. RSF, LassoCox and CoxBoost algorithms were further applied to evaluate the prognostic efficacy of TIIC-RNA.

Twenty algorithms including RSF, conditional random forest (CForest), LassoCox, elastic network regression (Enet), Ridge, gradient lift using regression trees (BlackBoost), parametric survival model regression (SurvReg), and conditional inference trees (CTree), CoxPH, ObliqueRSF, StepwiseCox, SurvivalSVM, generalized enhanced regression model (GBM), Ranger, cox model and partial least squares regression (PlsRcox) of related techniques, gradient lifting is performed using component linear models (GlmBoost), supervised principal components (SuperPC), akritas conditional nonparametric survival estimators (Akritas), CoxBoost, and recursive partition and regression trees (Rpart) to determine the most reliable model based on the comprehensive C-index. TIIC signature score was developed based on prognostic TIIC-RNAs by RSF algorithm. Cox regression and Kaplan–Meier analysis were performed using survival package.

### SMR and MR analysis

The GWAS data of ESCC patients were obtained from the gtca website (GCTA | Yang Lab (westlake.edu.cn). The manhattan map of GWAS was plotted using the CMplot package. Gene colocalization analysis was performed using SMR software based on eQTLGen data (EQTLgen-CIS-EQTLS) and GWAS data. Mendelian randomized (MR) analysis was conducted with “TwoSampleMR” R package to investigate the correlation between SNP sites of prognostic model genes and gastro-oesophageal reflux with ESCC.

### Statistical analysis

The correlation between two continuous variables was evaluated by the Pearson correlation coefficient. Categorical variables were compared by Chi-square test, and continuous variables were compared by Wilcoxon rank sum test or T test. A P-value less than 0.05 was considered statistically significant.

## Results

### Identification of TIIC-RNA at the single-cell level

**Fig. 1:**
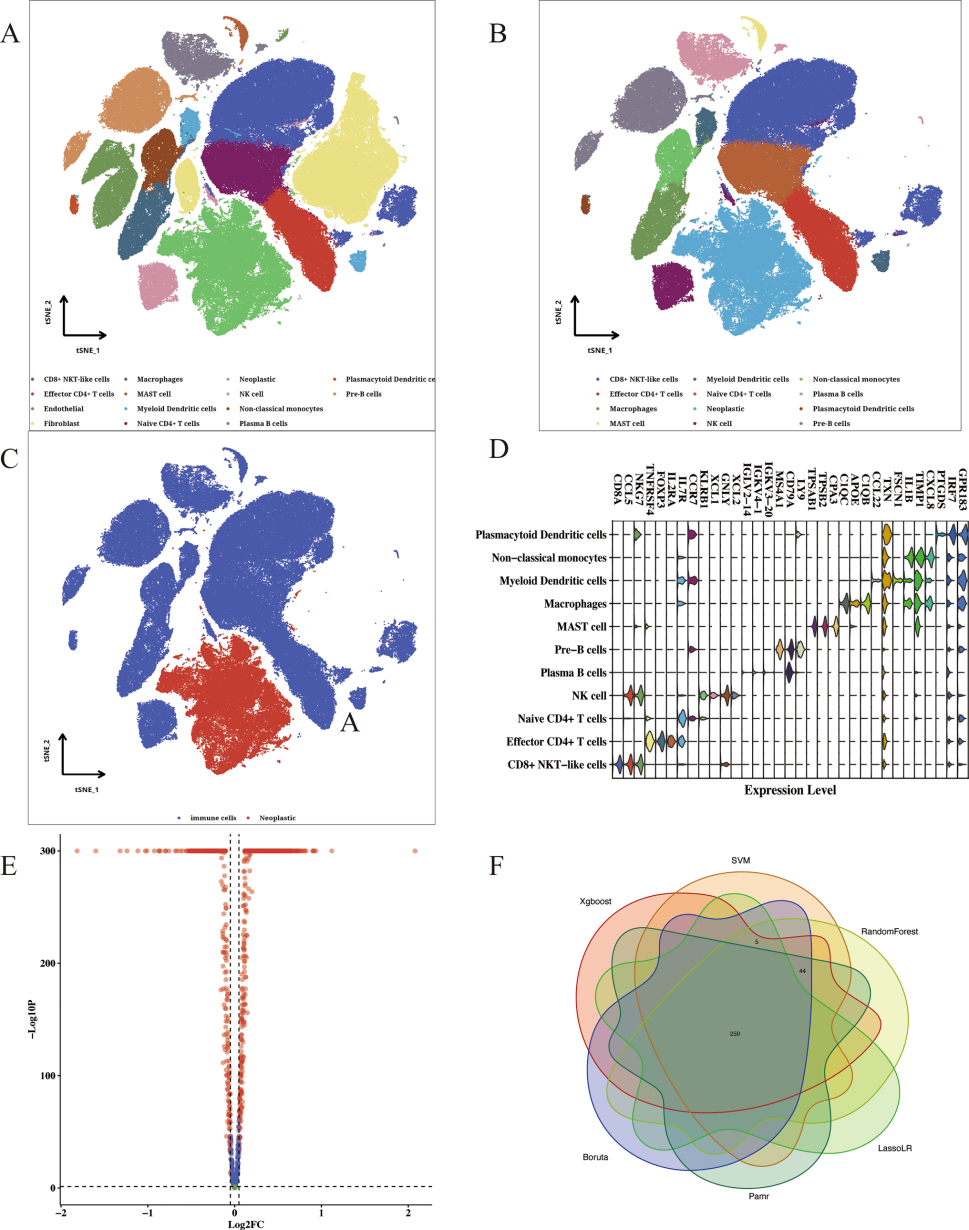
Identification of TIIC-RNA at the single-cell level. **A** t-SNE plot of cell clusters in tumor microenvironment. **B** t-SNE plot of immune cells types. **C** Diagram of the expression level of marker genes in each immune cell. **D** t-SNE plot of the distribution of immune cells and neoplastic cells. **E** Volcano plot of DEGs screen by immune cells and neoplastic cells. **F** Venn map of most valuable TIIC-RNAs selected by 6 algorithms

Performing dimension clustering analysis on the scRNA-seq dataset of ESCC, we identified all cell clusters in Fig. 1A. In the Fig. 1B, we selected 13 immune cell types for further analysis. Total 2,698 RNAs were defined as potential immune-related RNAs. 1,107 iuRNAs were were further identified when TSI < 0.45. We annotated marker gene with significant expression level in each immune cell (Fig. 1C). As presented in Fig. 1D, the distribution of immune cells and neoplastic cells was visualized. In addition, we identified DEGs between ESCC cells and immune cells in Fig. 1E. 299 significantly up-regulated DEGs were considered as TIIC-RNAs. After the integration of 6 machine learning algorithms, we finally obtained 250 valuable TIIC-RNA in Fig. 1F.

### Construction of TIIC signature score

**Fig. 2:**
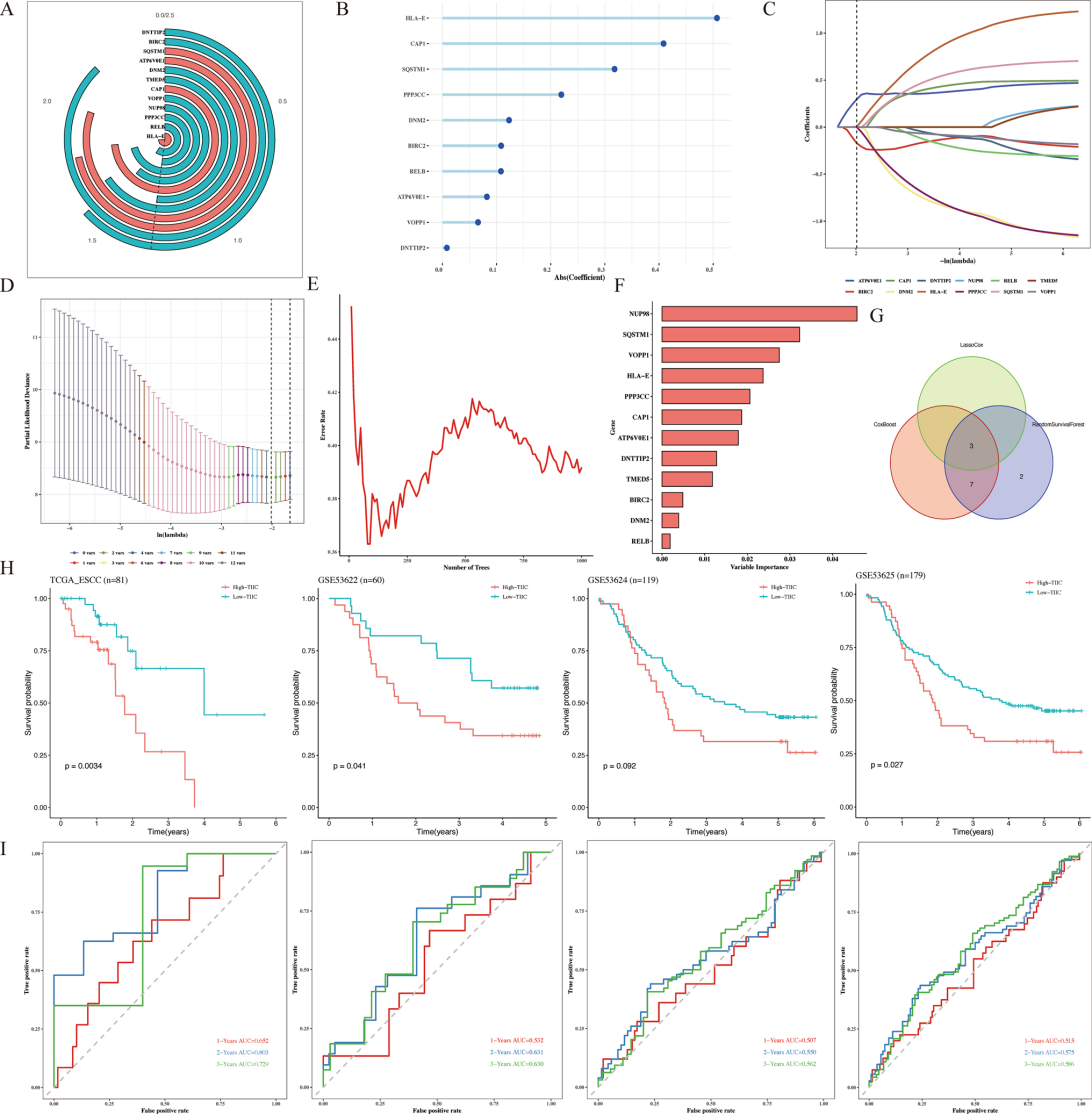
Construction of TIIC signature score.** A** Circle plot of 12 TIIC-RNAs by univariate Cox regression analysis. **B** Line map of 12 TIIC-RNAs evaluated by CoxBoost algorithm. **C**, **D** Diagrams of 12 TIIC-RNAs evaluated by LassoCox algorithm. **E**, **F** Diagrams of 12 TIIC-RNAs evaluated by Random Forest algorithm. **G** Venn plot of prognostic genes interacted by three algorithms. **H** K-M curves of OS of ESCC patients with high-TIIC and low-TIIC signature scores in different datasets. **I** ROC curves of 1, 2, 3 years of OS of ESCC patients with high-TIIC and low-TIIC signature scores in different datasets

We performed a univariate Cox regression analysis to investigate the prognostic value of TIIC-RNA for OS of ESCC patients. As it turned out, 12 TIIC-RNAs were identified in the TCGA dataset, including DNTTIP2, BIRC2, SQSTM1, ATP6V0E1, DNM2, TMED5, CAP1, VOPP1, NUP98, PPP3CC, RELB, and HLA-E (Fig. 2A). Three machine learning algorithms was conducted, containing CoxBoost (Fig. 2B), LassoCox (Fig. 2C, D), and Random Forest (Fig. 2E, F). We further determined three prognostic TIIC-RNA (Fig. 2G). We constructed the most reliable model based on a comprehensive C-index of externally validated datasets. TIIC signature score was developed based on prognostic TIIC-RNAs. In the TCGA-ESCC, GSE53622, GSE53624, and GSE53625 datasets, ESCC patients with high TIIC signature scores showed poorer survival outcomes in Fig. 2H. 1, 2, and 3 year- OS of ESCC patients were quantified by AUC values in TCGA-ESCC (0.652, 0.803, 0.729), GSE53622 (0.532, 0.631, 0.630), GSE53624 (0.507, 0.550, 0.562), and GSE53625 (0.515, 0.575, 0.586) (Fig. 2I).

### Prognostic value of TIIC signature score compared with clinical features

**Fig. 3:**
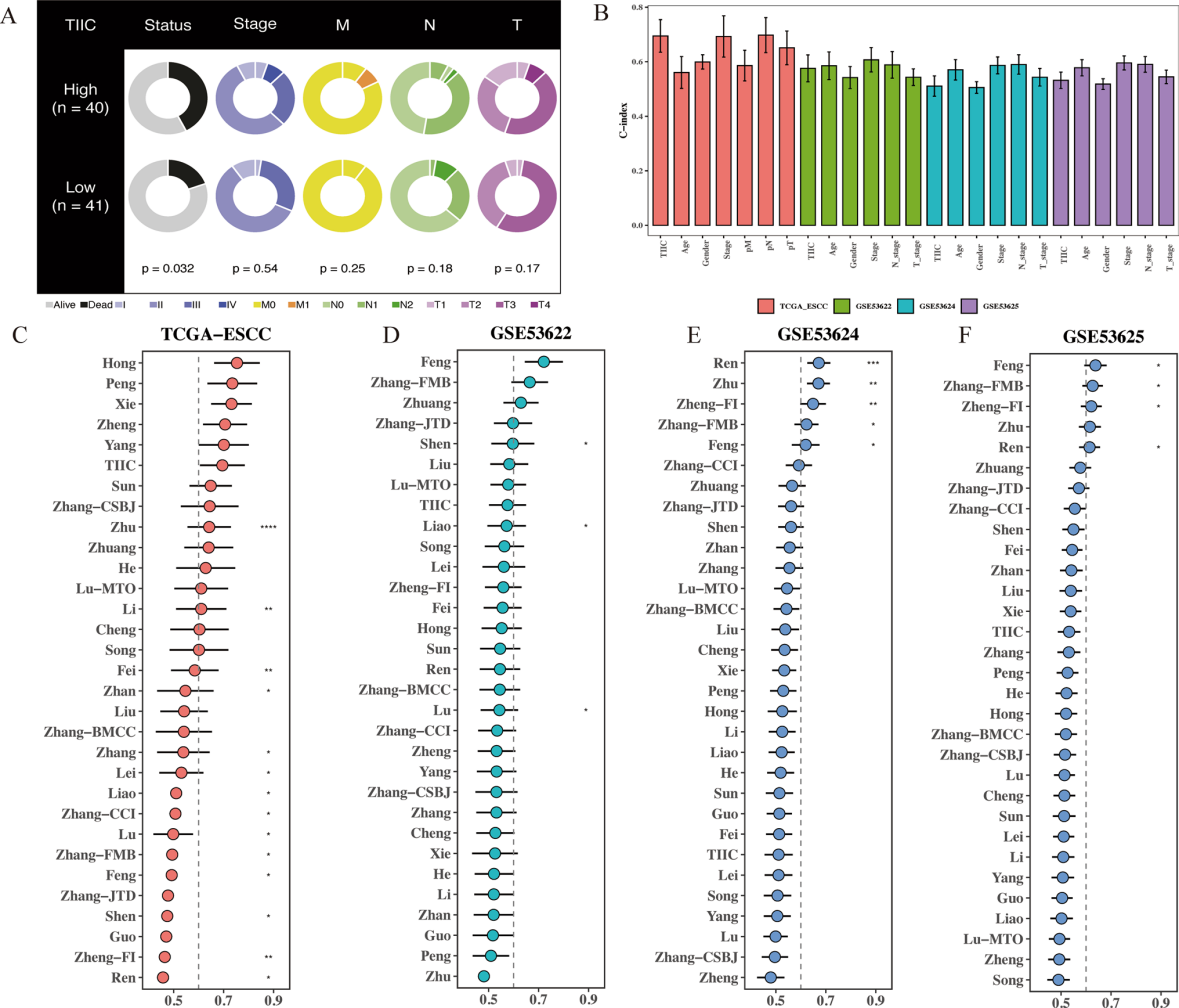
Prognostic value of TIIC signature score compared with clinical features. **A** Circos plot of the correlation of clinical characteristics between TIIC signature score groups. **B** Bar chart of the C-index of TIIC model and other clinical features in TCGA-ESCC, GSE53622, GSE53624 and GSE53625 datasets. **C**–**F** Line charts of the comparation between TIIC signature score and 30 published models in TCGA-ESCC, GSE53622, GSE53624 and GSE53625 datasets

In the TCGA dataset, TIIC signature score was significantly correlated with survival status (*p* = 0.032), but not with tumor stage and TNM staging system (Fig. 3A). In addition, in the TCGA dataset, TIIC signature score showed better performance in terms of age, sex, tumor stage, and TNM staging system with high C-index (Fig. 3B). To further validate the prognostic performance of TIIC signature score, we included 30 published prognostic models and compared the C-index in the TCGA-ESCC, GSE53622, GSE53624, and GSE53625 datasets (Fig. 3C-F). Our TIIC model shows better performance in TCGA-ESCC, GSE53622, GSE53624, and GSE53625 datasets than most other published models.

### Predicting biological mechanisms associated with TIIC signature score

**Fig. 4:**
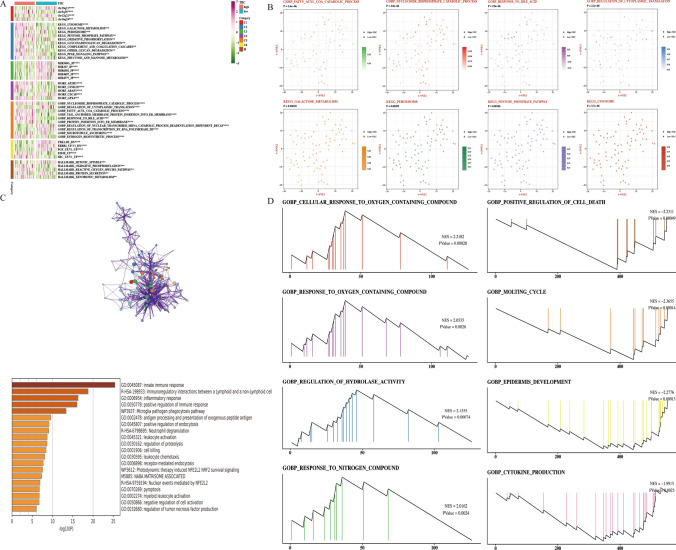
Biological characteristics of TIIC signature score. **A** Heat map of GSVA analysis in TIIC signature score groups based on MsigDB. **B** t-SNE plots of differences of GO and KEGG pathway between the TIIC scoring groups. **C** Enrichment analysis of differentially expressed genes in high-TIIC group. **D** GSEA graphs of GO and KEGG for high- and low-TIIC groups

TIIC signature score is strongly positively correlated with several biological pathways, mainly including KEGG_LYSOSOME, KEGG_GALACTOSE_METABOLISM, and KEGG_PEROXISOME (Fig. 4A). We selected 8 pathways from the GO-BP and KEGG databases that were significantly different between the two scoring groups and showed the ssGSEA scores corresponding to the pathways (Fig. 4B). We showed the enrichment results of up-regulated genes in the high-TIIC group on Metascape, which showed that they were related to immune response and inflammatory response (Fig. 4C). We exhibited GSEA results for key genes as follows: In the high-TIIC group, key genes are correlated with CELLULAR_RESPONSE_TO_OXYGEN_CONTAINING_COMPOUND, REGULATION_OF_HYDROLASE_ACTIVITY, RESPONSE_TO_NITROGEN_COMPOUND, Response_oxygen_containing_compound, and RESPONSE_TO_OXYGEN_CONTAINING_COMPOUND. In the low-TIIC group, key genes are related to MOLTING_CYCLE, EPIDERMIS_DEVELOPMENT, CYTOKINE_PRODUCTION, and POSITIVE_REGULATION_OF_CELL_DEATH (Fig. 4D).

### Correlation of TIIC signature score with immune-related features

**Fig. 5:**
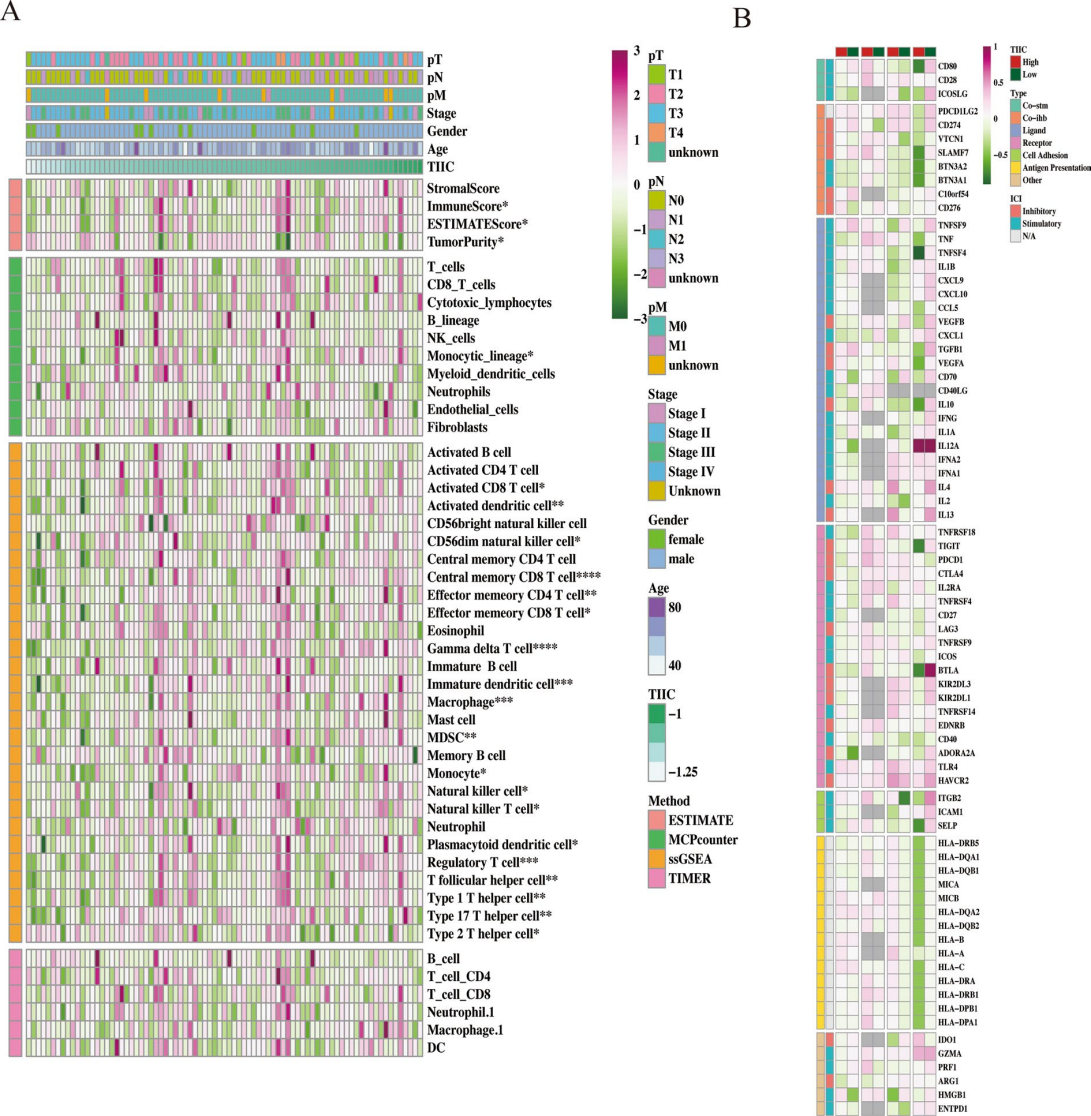
Correlation of TIIC signature score with immune-related features.** A** Heat map of correlation between TIIC signature score and immune infiltrated cells. **B** Heat map of correlation between TIIC signature score and immunomodulatory genes

As shown in Fig. 5A, we found that the activity of most immune cells decreased with the increase of TIIC score. At the same time, TIIC signature score showed difference in the expression levels of CD80, C10orf54, CD276, CD70 and methylation levels of CD80, CD28, CD274, CXCL1. There was also significant different correlation with the degree of CNV amplification of the immune checkpoint genes such as VTCN1, TNFSF9, and TNF, and CNV deletion of the immune checkpoint genes such as CD80, ICOSLG, PDCD1LG2, and CD274 (Fig. 5B).

### Verification of the prognostic value of TIIC signature score for immunotherapy response

**Fig. 6:**
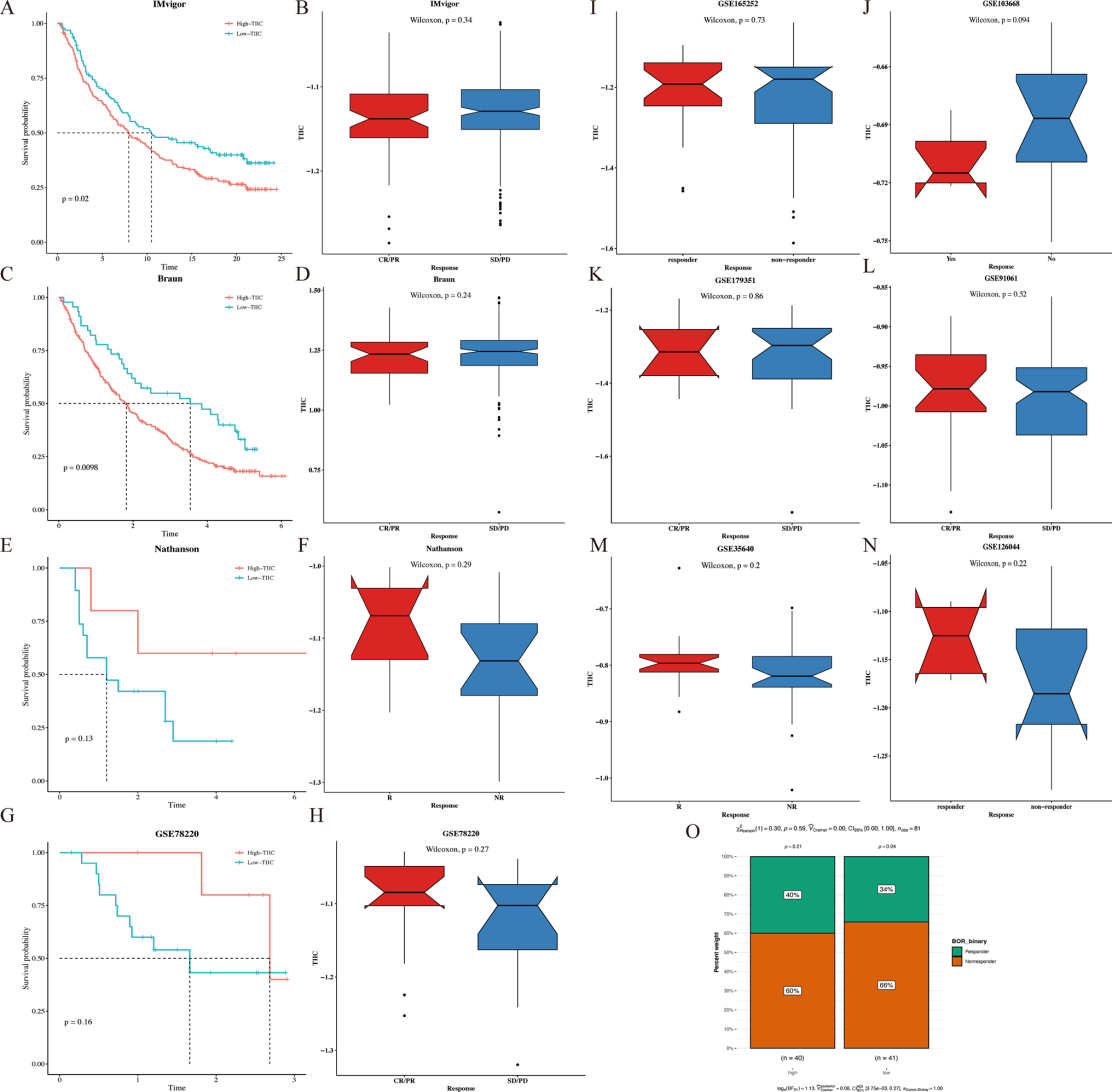
Verification of the prognostic value of TIIC signature score for immunotherapy response.** A**, **C**, **E**, **G** K-M curve of OS of ESCC patients in the IMvigor, Braun, Nathanson, and GSE78220 between TIIC signature score groups. **B**, **D**, **F**, **H** Comparison of response to immunotherapy of ESCC patients in the IMvigor, Braun, Nathanson, and GSE78220. **I**–**N** Comparison of response to immunotherapy of ESCC patients in the GSE165252, GSE103668, GSE179351, GSE91061, GSE35640, and GSE126044. **O** The percent weight of responder and non-responder between TIIC signature score groups

In the IMvigor dataset, patients with low TIIC feature scores showed better survival outcomes (*p* = 0.02, Fig. 6A) and both responded against PD-L1 immunotherapy (Fig. 6B). The same results were validated in the Braun dataset (*p* = 0.0098, Fig. 6C) with no difference in response to PD-L1 immunotherapy (Fig. 6D). However, in the Nathanson and GSE78220 datasets, survival outcomes and response to PD-L1 immunotherapy showed no significant different in both groups (Fig. 6E-H). In addition, patients with low TIIC signature score responded better to immunotherapy in GSE165252 (Fig. 6I), GSE103668 (Fig. 6J), and GSE179351 (Fig. 6K), while patients with high TIIC signature score responded better to immunotherapy in GSE91061 (Fig. 6L), GSE35640 (Fig. 6M), and GSE126044 (Fig. 6N). The TIDE results showed that in the TCGA dataset, a low proportion of patients responded in the low TIIC signature score group (*p* = 0.04, Fig. 6O).

### Predicting metabolic characteristics associated with TIIC signature score

**Fig. 7:**
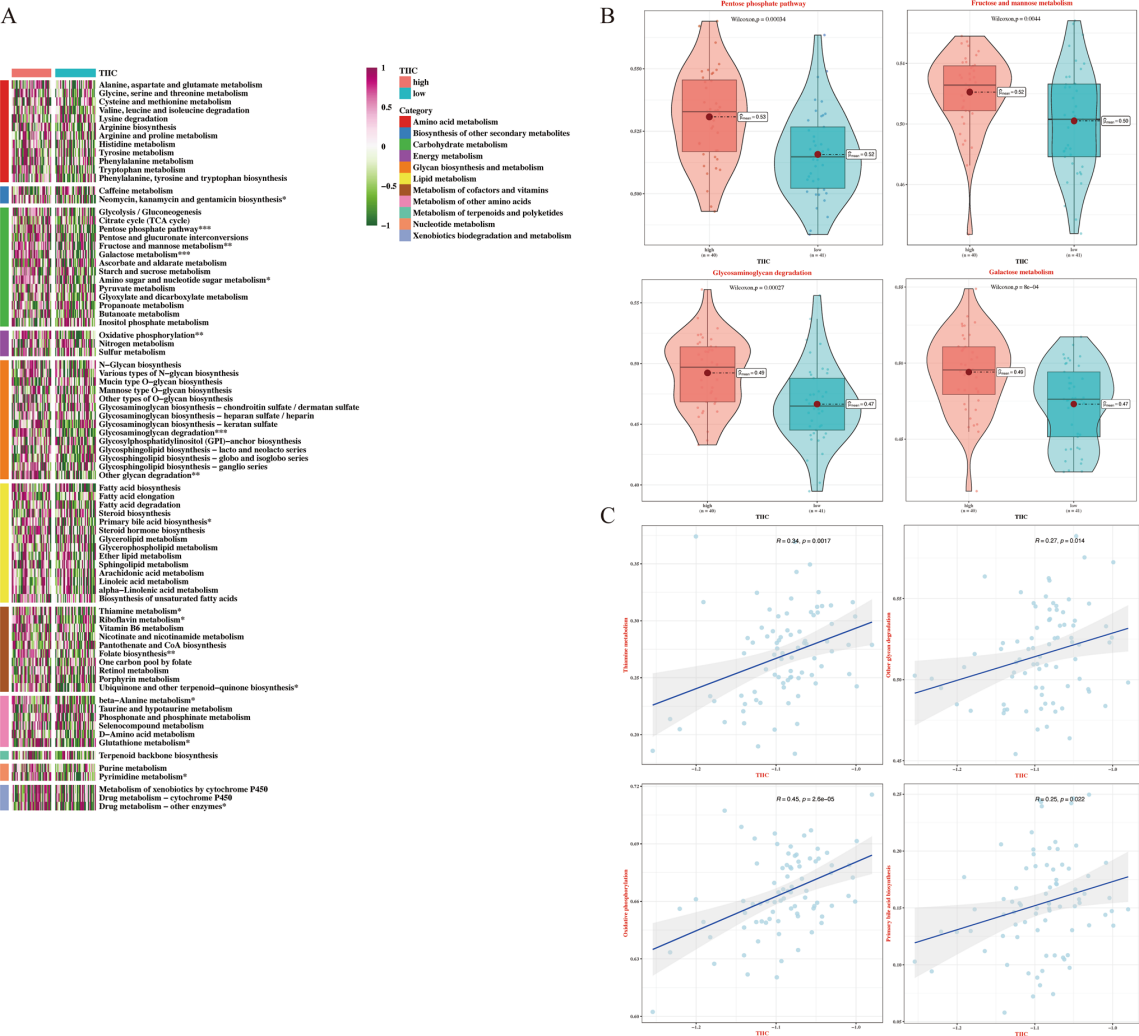
Metabolic characteristics of TIIC signature score in TCGA dataset. **A** Heat map of GSVA analysis of 11 metabolic pathway subgroups between two TIIC scoring groups based on KEGG. **B** Maps of differences in metabolic pathways between the two TIIC scoring groups. **C** Scatter diagram of correlation between TIIC signature score and metabolic pathways

To investigate a wide range of metabolic characteristics in the two TIIC signature score groups, GSVA was performed against metabolic pathways in the KEGG database. TIIC signature score was significantly correlated with many metabolic pathways (Fig. 7A). It is worth noting that pentose phosphate pathway, fructose and mannose metabolism, galactose metabolism and glycosaminoglycan degradation activation rates were significantly higher in the high TIIC signature score group compared to low TIIC signature score group (Fig. 7B). In addition, TIIC signature score was positively associated with oxidative phosphorylation, other glycan degradation, primary bile acid biosynthesis and thiamine metabolism (Fig. 7C).

### SNV mutation analysis and CNV analysis

**Fig. 8:**
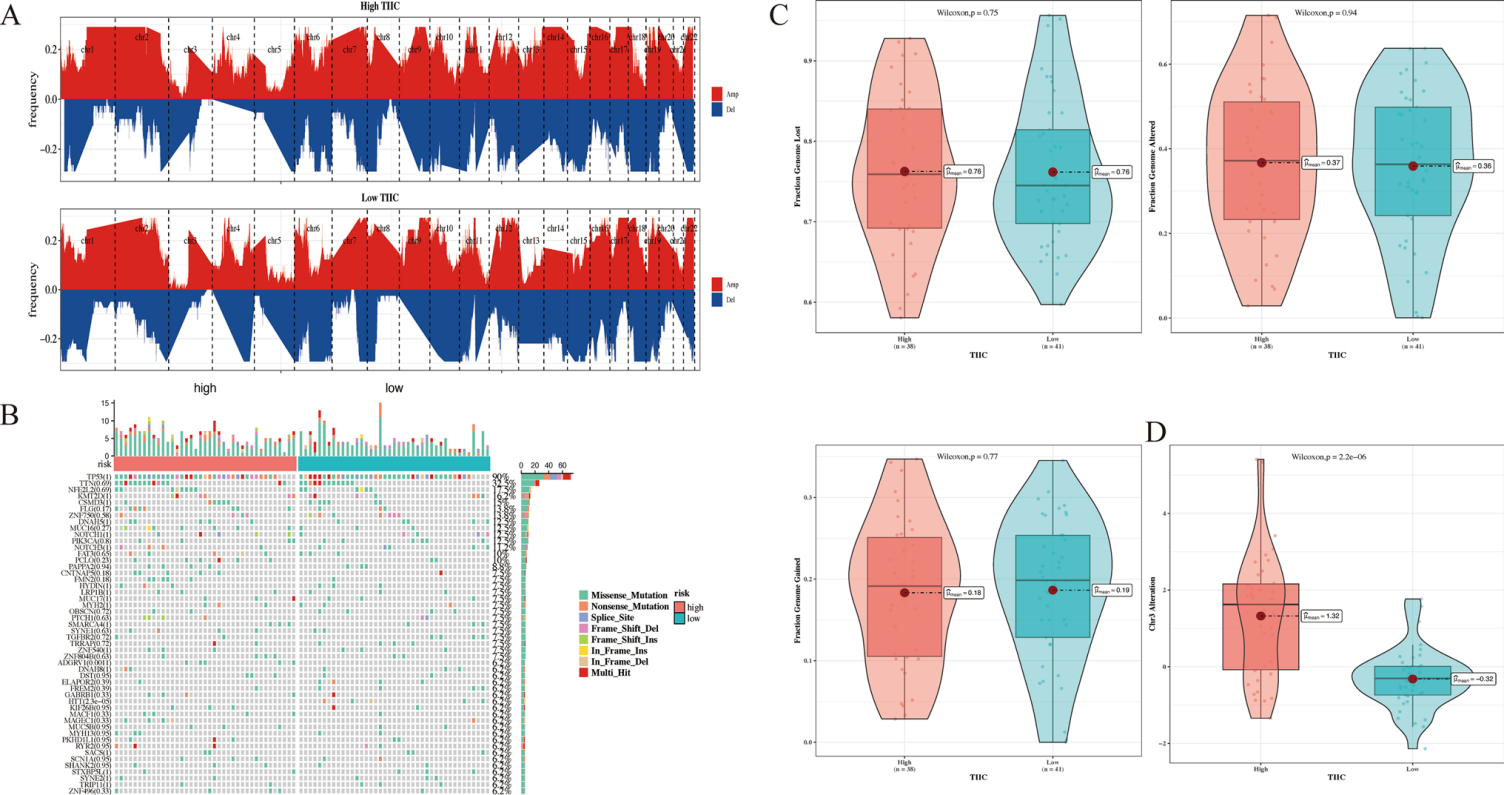
SNV mutation analysis and CNV analysis. **A** Diagram of chromosomal changes based on GISTIC 2.0 in two TIIC signature score groups. **B** Waterfall map of genomic mutation landscape in two TIIC signature score groups. **C** Diagram of changes of FGA, FGG, and FGL in both TIIC signature score groups. **D** Diagram of CNV mutations in chr3 between two TIIC signature score groups

Different frequencies of chromosomal changes were observed in the two TIIC score groups (Fig. 8A). The waterfall map shows the mutations of top 50 genes in both groups. It can be seen that TP53 (90%), TTN (32.7%) and NFE2L2 (17.5%) are genes with high mutation rates, among which ADGRV1 (6.2%) and HTT (6.2%) have significant differences between the two groups (Fig. 8B). There is no statistically significant change of fraction genome altered (FGA), fraction genome gained (FGG), and fraction genome lost (FGL) between high- and low-TIIC signature score groups in Fig. 8C. CNV mutations in chr3 were significantly different between the two groups (*p* = 2.2e-06, Fig. 8D).

### SMR and MR analysis

**Fig. 9:**
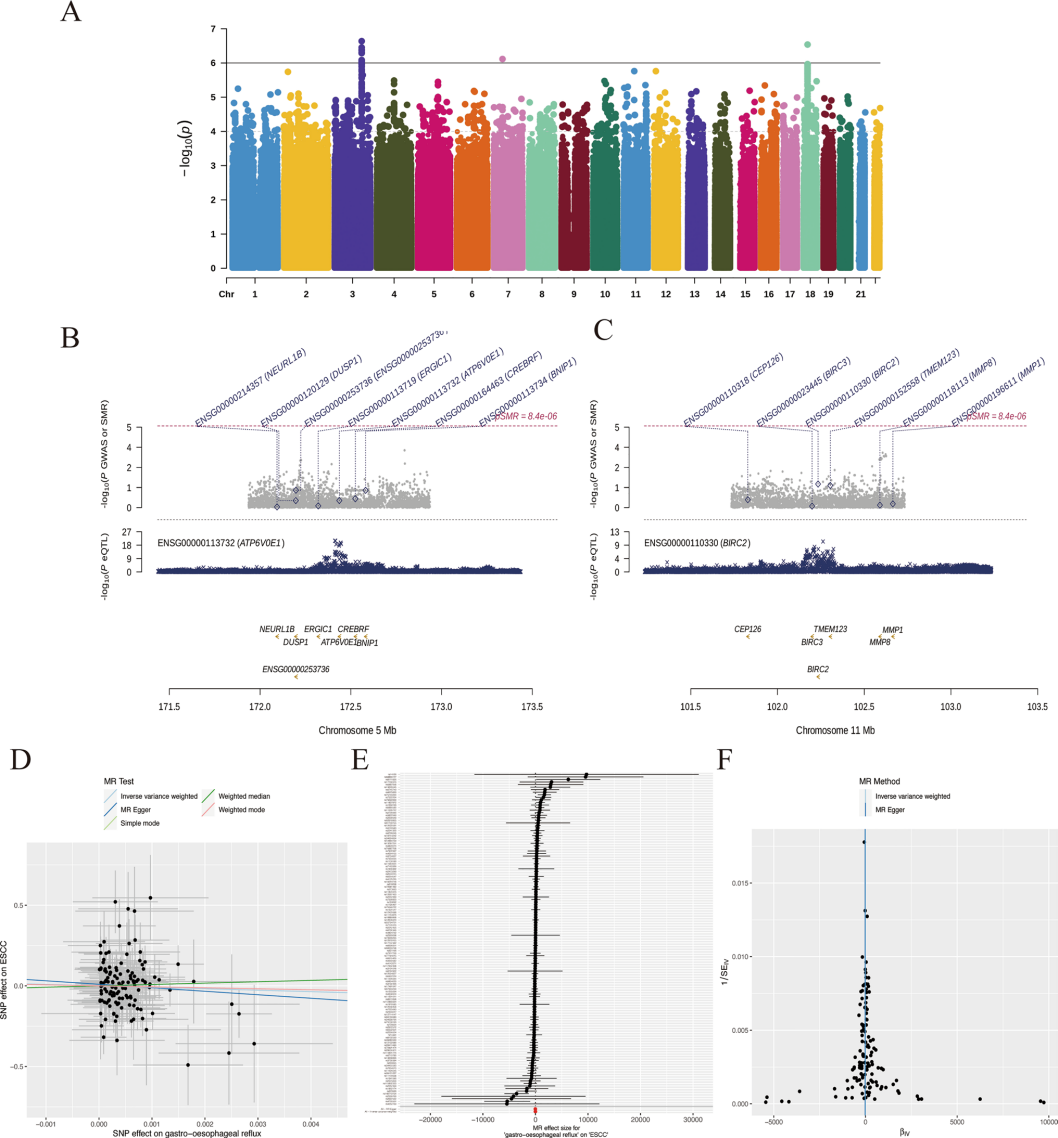
SMR and MR analysis.** A** Manhattan map of GWAS data for ESCC. **B**, **C** Gene colocalization analysis of two prognostic model genes (ATP6V0E1, BIRC2). **D**–**F** Scatter plot, funnel plot, and forest plot of MR analysis between gastro-oesophageal reflux and ESCC

GWAS data for ESCC were visualized using the Manhattan chart (Fig. 9A). Gene colocalization analysis was performed, showing two prognostic model genes (ATP6V0E1 and BIRC2) in Fig. 9B, C. MR analysis found that gastro-oesophageal reflux were not significantly associated with ESCC based on SNP of TIIC-RNA gene, but rs148710154 and rs75146099 SNP sites had a significant correlation between them (the result of a single SNP can be seen in gastro-oesophageal reflux_to_cancer_MR_snps_result.xlsx file, Fig. 9D-F).

## Discussion

ESCC is a globally prevalent form of malignancy and ranks as the ninth most severe malignant cancer [2]. Despite advancements in treatment approaches and notable outcomes, the prognosis for ESCC patients remains unsatisfactory [16]. The TNM staging system, while widely used, does not account for the molecular heterogeneity of ESCC. Consequently, there is an urgent need to establish a precise prognostic prediction method that aligns with the present treatment and diagnosis of ESCC. This study constructed a novel prognostic signature score capable of accurately forecasting the prognosis for ESCC, named as TIIC signature score. Our findings suggest that the TIIC signature score not only outperforms the TNM system in terms of predictive accuracy but also provides molecular insights into the tumor microenvironment.

TME is a multifaceted milieu in which tumor develops and progresses, comprising diverse tumor cells, immune cells, and stromal cells [17]. Stromal cells, in comparison to tumor cells within the TME, exhibit genetic stability and are regarded as a potentially valuable biomarker for cancer therapy [18]. Furthermore, immune cells within the TME play a crucial role in the initiation and spread of tumors, and their significance in the effectiveness of immunotherapy is underscored by the potential mechanisms linked to Treg-macrophage interactions with ESCC. T cells play a crucial role as targets of checkpoint inhibitors and significantly impact the treatment outcome of immunotherapy in tumor therapy [19]. Recent investigations have unveiled that T cells in treatment of ESCC consistently exhibit dysfunctional and exhausted characteristics, thereby exacerbating the decline in antitumor immune status in locally advanced ESCC [20]. Moreover, scRNA-seq has revealed the presence of diverse signaling pathways originating from heterogeneous stromal cells, which contribute to the distinct traits observed in immune cells within ESCC [21]. Consequently, the analysis of immune cells within the TME holds the potential to provide novel insights in the field of immune research.

In this study, we identified TIIC-RNA that can precisely forecast the prognosis of ESCC patients. A new prognostic score for ESCC was constructed for the first time which was significantly correlated with survival status of the patients. Our TIIC signature score had excellent performance than other existed models in predicting ESCC. It demonstrated the possible importance of tumor-infiltrating immune cell in the TME of ESCC tissues. Numerous studies have also highlighted the significant role of TIICs in tumor progression [22]. Notably, the presence of high TIIC expression within the TME has been associated with unfavorable clinical prognostic outcomes in certain cancers, including ESCC. This research also found enrichment of lysosome, galactose metabolism, and peroxisome signaling pathways in the high-score group. ESCC patients in high-score groups exhibited correlations with CNV amplification or deletions of the immune checkpoint genes, whereas ESCC patients had varying sensitivity to immunotherapy and chemotherapeutic agents. CNV is a significant type of genomic structural variation. CNV is characterized as the the changing number of copies of a specific DNA segment (> 1 kb in length) in comparison to the controls [23]. CNVs can induce phenotypic changes through various mechanisms, such as modifying the copy number of gene or altering nearby or distant DNA regulatory regions that can impact the transcription [24]. In recent studies, it has been discovered that common CNVs with a minor allele frequency of at least 5% are implicated in various genetic and developmental disorders [25]. Consequently, investigating CNVs associated with cancer may lead to the identification of genes that take part in the underlying mechanisms of tumorigenesis.

Furthermore, the prognostic significance of the TIIC score model was validated in both the training and validation cohorts to enhance its utility. Our findings demonstrated a clear distinction in overall survival rates between scoring groups. Moreover, the TIIC signature score exhibited the ability to predict survival outcomes in patient groups, akin to the TNM staging system, suggesting its potential as a prognostic tool that could complement the current TNM staging method. In this study, we investigated the correlation between the developed prognostic models and the immune infiltration of tumors, response to immunotherapy, and survival outcomes. The findings of this research will provide the advancement of prognosis and immunotherapeutic strategies for ESCC, as well as personalized care.

In addition, we found different frequencies of chromosomal changes in two TIIC signature score groups, indicating the genomic variations in ESCC. Gene colocalization analysis was performed, showing two prognostic model genes (ATP6V0E1 and BIRC2). The ATP6V0E1 gene encodes a key subunit of V-ATPase, an enzyme essential for cancer cell survival [26]. Studies suggest that ATP6V0E1 may regulate tumor cell metabolism in ESCC through oxidative phosphorylation and cuproptosis pathways. Furthermore, the overexpression of ATP6V0E1 may be associated with metabolic dysregulation and immune cell suppression within the tumor immune microenvironment [27]. The BIRC2 gene, on the other hand, is involved in regulating inhibitors of apoptosis proteins and plays a critical role in suppressing tumor cell apoptosis and promoting immune evasion [28]. Recent studies have shown that BIRC2 is closely linked to the expression of the CXCL9 chemokine, which recruits T cells and NK cells, thereby modulating immune regulation within the tumor microenvironment [29]. MR analysis found that rs148710154 and rs75146099 SNP sites of TIIC-RNA gene had a significant correlation between them gastro-oesophageal reflux and ESCC. These findings offered the genomic evidence for the mechanisms of development of ESCC. However, ther remain some limitations in this research. First, the potential impact of the small sample size on the results cannot be overlooked. Furthermore, our conclusions are derived from in analyses of RNA expression data obtained from public datasets. It is necessary for confirmation through experiment and clinical studies. The functional significance of these TIIC-RNAs was needed further study to confirm the enrichment of specific pathways and the prognostic landscape.

## Conclusion

TIIC signature score was the first time developed which provided a novel strategy and guidance for the prognosis and immunotherapy of ESCC. It also gave the evidence in the important role of immune cells from the TME in the treatment of cancers.

## Data Availability

The datasets generated during and/or analyzed during the current study are available from the corresponding author on reasonable request.
